# Bromide of Potassium as a Substitute for the Iodide

**Published:** 1847-05

**Authors:** 


*Bromide of Potassium as a substitute for the Iodide.*—The low price of the bromide compared with that of the iodide of potassium has induced M. Ricord to substitute the former for the latter in the treatment of secondary syphilitic affections. The dose of the bromide is the same as of that of the iodide of potassium. It has produced the same therapeutical effects but more slowly.—*Journal de Pharmacie.*

